# Myocardial Work Indices in Patients Recently Recovered from Mild-to-Moderate COVID-19

**DOI:** 10.3390/jcm13144090

**Published:** 2024-07-12

**Authors:** Rafał Dankowski, Wioletta Sacharczuk, Julita Fedorowicz, Małgorzata Małek-Elikowska, Stefan Ożegowski, Artur Baszko

**Affiliations:** 2nd Department of Cardiology, Poznan University of Medical Sciences, 60-485 Poznan, Poland; wioletta.sacharczuk@ump.edu.pl (W.S.); jfedorowicz@ump.edu.pl (J.F.); abaszko@ump.edu.pl (A.B.)

**Keywords:** myocardial work, echocardiography, COVID-19, myocardial injury, high-sensitive troponin I, peak strain dispersion

## Abstract

**Background/Objectives**: Persistent cardiovascular issues are common in COVID-19 survivors, making the detection of subtle myocardial injuries critical. This study evaluates myocardial work (MW) indices in patients recently recovering from mild-to-moderate COVID-19. **Methods:** A total of 105 recently recovered COVID-19 patients (who had a mean age of 52 years) underwent comprehensive laboratory testing and advanced echocardiographic assessments. The median time since their COVID-19 infections was 56 days (IQR: 42–71). The cohort was stratified based on high-sensitive troponin I (hs-TnI) levels: undetectable versus detectable. The echocardiographic analysis utilized pressure-strain loops to evaluate MW indices. **Results**: Detectable hs-TnI levels were observed in 42% of patients. The median values of MW indices for the entire group were slightly below normal values: global work index (GWI)—1834 mmHg% (IQR 1168–2054 mmHg%), global constructive work (GCW)—2130 mmHg% (IQR 2010–2398 mmHg%), global wasted work (GWW)—119 mmHg% (IQR 78–175 mmHg%), and global work efficiency (GWE)—94% (IQR 92–96%). Patients with detectable hs-TnI had higher GWW (168 vs. 97 mmHg%, *p* < 0.005) and lower GWE (93% vs. 95%, *p* < 0.005). In multiple regression analysis, strain dispersion (PSD) was the sole predictor for GWW (β = 0.67, *p* < 0.001), while for GWE, PSD (β = −0.67, *p* < 0.001) and LVEF (β = 0.16, *p * = 0.05) were significant predictors. **Conclusions:** Among patients recently recovering from mild-to-moderate COVID-19, elevated hs-TnI levels are linked with a reduction in GWE and an increase in GWW. PSD is an important predictor of myocardial inefficiency and wasted work. In this group, disruptions in the timing and coordination of cardiac muscle contractions may play a key pathophysiological role in reducing the efficiency of the heart’s performance.

## 1. Introduction

The COVID-19 pandemic, caused by the SARS-CoV-2 virus, has profoundly impacted global health. While it is primarily a respiratory illness, COVID-19 can also affect other organ systems, including the cardiovascular system [1]. 

Cardiac injury has been observed in a significant proportion of patients hospitalized with COVID-19 [2]. This condition is associated with a higher risk of mortality and may reflect direct myocardial injury caused by the virus, systemic inflammation, or exacerbation of underlying cardiovascular disease [3]. Persistent cardiovascular effects have been observed several months after recovery, indicating sustained cardiac stress or injury [4].

Although some studies are available [5,6,7,8,9], the research focusing on patients who did develop non-severe COVID-19 receives less attention compared to those involving critically ill patients. This group is less studied compared to those who experienced severe illness requiring hospitalization. Furthermore, fewer studies have evaluated this cohort immediately after recovery from the disease compared to the more extensively studied group with long COVID-19 [7]. This disparity in research underscores the need for more targeted studies to understand the cardiovascular implications in patients with mild-to-moderate COVID-19 [10].

The processes underlying myocardial injury in the context of COVID-19 recovery are not well understood [11]. The pathophysiological mechanisms driving myocardial dysfunction post-COVID-19 remain poorly characterized, posing challenges in determining their relative contribution and determinants, particularly in patients recovering from the disease. This lack of understanding highlights the need for comprehensive assessments to unravel the long-term implications of COVID-19 on cardiovascular health.

Recently introduced noninvasive echocardiographic assessment of myocardial work (MW), derived from noninvasive pressure-strain loops [12], provides a more comprehensive understanding of myocardial performance than traditional measures of systolic function [13]. This method considers the afterload, thus providing a load-independent measure of myocardial function [14]. Assessment of MW could be particularly relevant in the context of COVID-19, where the disease process may persist beyond the acute phase, potentially influencing a patient’s hemodynamic status even after the resolution of the acute infection [15]. In the literature, only a few studies assess myocardial work in patients following recovery from COVID-19 [16,17,18].

Thus, this study aims to evaluate myocardial work indices and their correlations with echocardiographic parameters and markers of myocardial injury in patients who have recently recovered from acute COVID-19. We hypothesize that patients with persistently elevated troponin levels will exhibit alterations in myocardial work indices, reflecting persistent myocardial dysfunction post-recovery from the acute phase of COVID-19.

## 2. Materials and Methods

This research was conceived as a single-site, cross-sectional investigation. Patient examinations were conducted at the Second Department of Cardiology, St. John Paul II HCP Hospital in Poznan, Poland. Primary healthcare providers in Poznan referred outpatients who had recently recovered from COVID-19. The patient assessments were conducted between 9 February and 16 April 2021. The criteria for inclusion were as follows: age above 18 years; confirmation of COVID-19 infection by SARS-CoV-2 real-time PCR using nasopharyngeal swabs at least 28 days prior to the assessment. Our study encompassed patients treated at home or hospitalized due to COVID-19. At the time of the study, none of the patients had received a COVID-19 vaccination.

During the comprehensive assessment, each patient’s medical history was meticulously reviewed, encompassing details about previous illnesses, hospitalizations related to COVID-19, treatments received, and any complications experienced during hospitalization. The physical examination of patients involved the evaluation of vital signs, including blood pressure and resting heart rate, as well as weight and height measurements. Body mass index (BMI) was calculated using the formula BMI = weight/height^2^ (kg/m^2^). Resting 12-lead electrocardiograms (ECGs) were recorded in all patients using a Philips PageWriter TC20 electrocardiograph. ECGs were recorded according to the usual clinical practice.

The laboratory panel included a complete blood count, high-sensitive cardiac troponin (hs-TnI), C-reactive protein (hsCRP), D-dimer, N-terminal pro-B-type natriuretic peptide (NT-proBNP) and serum creatinine. The estimated glomerular filtration rate (eGFR) was calculated using the Chronic Kidney Disease Epidemiology Collaboration (CKD-EPI) formula, which incorporates serum creatinine, age, gender, and race for accurate assessment [19]. The detection cutoff for hs-TnI was >1.6 pg/mL. Significantly elevated levels of high-sensitive hs-TnI were defined as concentrations exceeding the general population’s 99th percentile upper reference limit [20] and set at 15.6 pg/mL. The upper limit for NT-proBNP was defined as 125 pg/mL.

We analyzed the entire group and subgroups based on troponin levels: Trop−, with hs-TnI levels below the detection threshold, and Trop+, with detectable levels of hs-TnI.

### 2.1. Transthoracic Echocardiography

Echocardiography examinations were performed using the Vivid S70N ultrasound system (GE Healthcare, Horten, Norway, software version 203). Patients were positioned in the left lateral decubitus position, and cardiac images were acquired from standard echocardiographic views, including the parasternal long-axis, three parasternal short-axes, apical four-chamber, apical two-chamber, and apical three-chamber views.

Conventional echocardiographic measurements were performed according to the current recommendations of the European Association of Cardiovascular Imaging (EACVI) and the American Society of Echocardiography (ASE) [21]. Mitral Valve (MV) parameters, including MV E-wave Velocity, MV A-wave Velocity, MV Deceleration Time, Mitral Valve E/A Ratio, E′, and E/E′, were assessed using Doppler and Tissue Doppler Imaging techniques. Left Ventricular Ejection Fraction (LVEF) was calculated using the biplane Simpson’s method. The segmental wall motion of the left ventricle was assessed using a 16-segment model.

### 2.2. Global Longitudinal Strain and Myocardial Work Analysis

Global longitudinal strain and myocardial work were analyzed in two stages. Initially, the global longitudinal strain was assessed using speckle-tracking echocardiography performed on an echocardiography machine. Subsequently, the data were transferred to a workstation equipped with EchoPAC software, version 204, for further analysis of myocardial work by the dedicated application. RD and WS, experienced in echocardiographic studies, performed the acquisition and preliminary analysis of GLS. The researchers remained blinded to the clinical data during these stages to avoid bias in the analysis. RD carried out a subsequent offline analysis of MW on most patients. If uncertain, the analysis was performed jointly by RD and WS to ensure accuracy and consistency.

**Figure 1:**
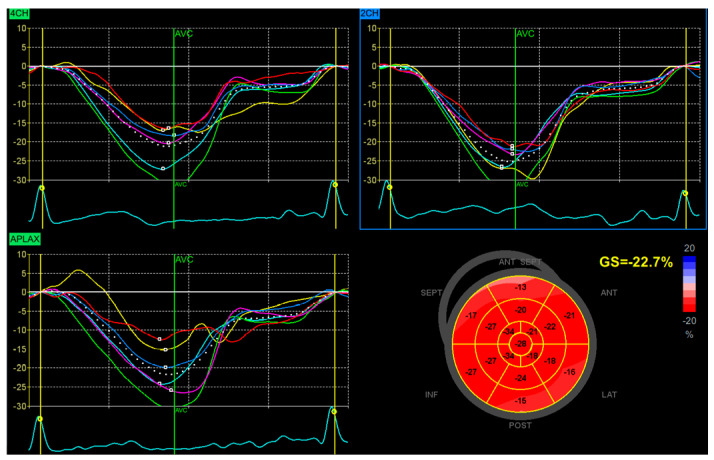
Presentation of Results of Global Longitudinal Strain Analysis. The **Top Left** Panel, **Bottom Left** Panel, and **Top Right** Panel display the strain curves for the segments of the left ventricle in the four-chamber view (4CH), the three-chamber (apical long-axis) view (APLAX), and the two-chamber view (2CH), respectively. Each segment is indicated by a different colored line (red, green, yellow, blue, magenta, cyan), representing individual measurements of strain over time. The dotted line indicates the average strain curve generated from the six-segment strain curves. The Bottom Right Panel presents a planar map (bull’s eye) of left ventricular strain. This visualization summarizes the segmental and global strain values. Each segment is color-coded according to the level of strain, with red indicating contraction and blue indicating relaxation. The Global Strain (GS) value is provided. GS represents the average peak strain across all segments of the left ventricle.

**Figure 2:**
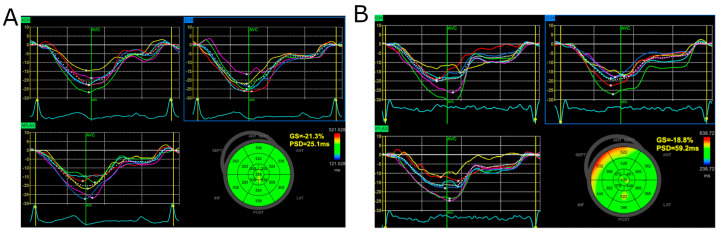
Presentation of Results of Peak Strain Dispersion (PSD) Analysis. Panel (**A**) Shows an Example of Normal PSD, While Panel (**B**) Shows Greater PSD Reflecting Less Homogeneous Contraction of the Left Ventricle. Detailed Description of Panels (**A**) and (**B**): The descriptions for the **Top Left**, **Bottom Left**, and **Top Right** Panels are similar to those in Figure 1. The Bottom Right Panel presents a planar map (bull’s eye) of Time to Peak (TTP) Strain for the left ventricle (milliseconds). Each segment is color-coded according to the time to peak strain: green indicates normal timing, while yellow to red indicates varying degrees of delayed contraction. The Global Strain (GS) and Peak Strain Dispersion (PSD) values are provided. PSD measures the temporal dispersion of peak strain values across different segments, indicating the synchrony of ventricular contraction.

The global longitudinal strain was assessed following the current Consensus Document of the EACVI/ASE/Industry Task Force [22]. The measurements were performed using a dedicated application implemented in the echocardiography machine—Automated Function Imaging (AFI). We obtained two-dimensional cine loops (three cardiac cycles) of the three apical views (four-chamber, two-chamber, and three-chamber views) for the analysis. The images were optimized for sector and depth, and the frame rate was set to 50–70 frames per second. The AFI software automatically suggests setting the region of interest. If the tracking was not optimal, the echocardiographer made the manual correction. Results of the speckle tracking analysis are presented as a global longitudinal strain value (negative value, %) and in the form of a planar map (Bull’s eye) and strain curves for each view (Figure 1). Peak strain dispersion was calculated automatically as a standard deviation of time to peak longitudinal strain for all segments [23] (Figure 2).

Assessment of myocardial work was based on a pressure-strain loop analysis [14]. This non-invasive method utilizes global longitudinal strain analysis and non-invasive measurement of blood pressure using an arm cuff, and it requires the determination of valvular event timing [24]. 

Since the myocardial work assessment requires arterial blood pressure, it was measured using an arm cuff and an automated device during the strain analysis.

**Figure 3:**
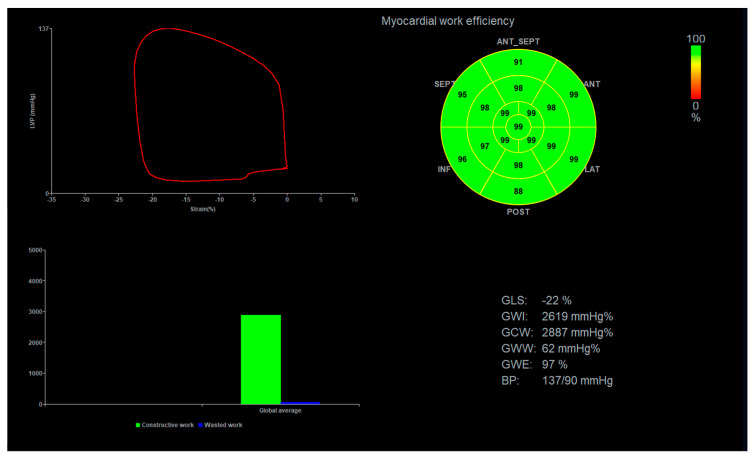
This figure shows an example of the results of Myocardial Work analysis.

The closure time of the mitral and aortic valves was set from a 2D apical three-chamber view. The analysis was performed semi-automatically using a dedicated application. The following myocardial work parameters were obtained: Global Constructive Work (GCW), representing the energy consumed during myocardial contraction; Global Wasted Work (GWW), reflecting energy dissipation during relaxation; Global Work Efficiency (GWE), indicating the proportion of constructive work relative to the total work; and Global Work Index (GWI), a comprehensive measure incorporating both constructive and wasted work (Figure 3).

All patients signed informed consent before entering the study. The Bioethics Commission of Poznan University of Medical Sciences approved the study. The study complies with the requirements of the Declaration of Helsinki.

### 2.3. Statistical Analysis

The data distribution was assessed for normality using the Shapiro-Wilk test. As most of the analyzed variables did not meet the criterion for a normal distribution, all data are presented as the median and interquartile range (25–75th percentile). Categorical variables are expressed as numbers and percentages. The Mann-Whitney U test was applied for group comparisons. Categorical data were compared using the Chi-square test. Correlations were evaluated using the Spearman correlation coefficient. Multiple regression was used to examine the association between myocardial work indices and examined anthropometric, laboratory, and echocardiographic parameters. Statistical significance was considered at *p* < 0.05. 

Interobserver and Intraobserver Variability: To evaluate the intra- and interobserver variability of GLS, PSD, and MW indices, a comparison of intra- and interobserver studies was performed according to the Intraclass Correlation Coefficient (ICC). For intraobserver, the same observer (RD) reevaluated 20 randomly selected examinations. For the estimate of interobserver variability, two independent observers (RD, WS) have marked the same 20 examinations. The ICC was calculated under the conditions of absolute agreement. For the intraobserver variability, the reliability of single evaluations was estimated; in the case of the interobserver variability, the estimation was performed for averages of k evaluations.

Sample Size Calculation: The sample size was determined by comparing means between the Trop+ and Trop− subgroups to achieve 5% significance (α = 0.05) and 80% power (β = 0.2). The calculated sample size based on the observed mean differences and standard deviations was 22 patients in each group for GLS, 35 patients in each group for PSD, 25 in each group for GWW, and 39 in each group for GWE. Given the observed differences between the groups, our sample size was underpowered for the assessment of GWI and GCW.

All analyses were carried out using the Statistica data analysis software system, version 13 (TIBCO Software Inc., Palo Alto, CA, USA, 2017), except sample size calculation, interobserver analysis, and intraobserver variability, which were analyzed by MedCalc^®^ Statistical Software, version 22.026. (MedCalc Software Ltd., Ostend, Belgium; https://www.medcalc.org; (accessed on 1 May 2024)).

## 3. Results

Out of the entire cohort of 105 patients, blood samples were unavailable for three patients. Due to suboptimal imaging quality, GLS and MW analyses were impossible in six patients. Additionally, we excluded three patients with significantly outlier troponin I concentrations (21.6, 31.7, and 48.2 pg/mL), exceeding the general population’s 99th percentile upper reference limit. Consequently, laboratory data were analyzed for 99 patients, and deformation analysis was carried out in 97 patients.

Basic characteristics of laboratory, electrocardiographic, and echocardiographic data of the entire study group are presented in Table 1. The analyzed group consisted of 102 patients, 57 women (56%), with a mean age of 52. The average time (IQR) from the diagnosis of COVID-19 was 56 (42–71) days.

Twenty-six patients (26%) were hospitalized. None of the patients were in a critical stage of COVID-19, requiring admission to the intensive care unit, intubation, or support from heart-lung machines or extracorporeal membrane oxygenation (ECMO). All patients were treated according to the current guidelines, reflecting the standard care practices aimed at mitigating the progression of COVID-19 in hospitalized patients [25]. 

For one patient, data regarding hospital treatment were unavailable. During hospitalization, all patients received supplemental oxygen therapy. Additionally, 3 patients (12%) were treated with AIRVO 2 High Flow Nasal Cannula Therapy, and 1 patient (4%) received Continuous Positive Airway Pressure (CPAP). Antibiotic therapy was administered to 22 patients (88%). All hospitalized patients received prophylactic anticoagulation with low-molecular-weight heparins (LMWH). Systemic steroid therapy was provided to 22 patients (88%). Convalescent plasma was administered to 13 patients (52%), tocilizumab to 4 patients (16%), and Remdesivir to 12 patients (48%). 

Among the patients who did not require hospitalization (n = 79), non-steroidal anti-inflammatory drugs (including paracetamol and acetylsalicylic acid) were administered to 66 individuals (83.5%). Antibiotics were given to 18 patients (22.8%), oral steroids to 4 patients (5.1%), and low-molecular-weight heparins to 5 patients (6.3%). There were no patients in this group who required home oxygen therapy.

Electrocardiograms. One patient did not have an ECG recorded, so the analysis was conducted for 104 patients. All patients were in sinus rhythm, and none of the patients had a previous history of atrial fibrillation. Bradycardia (heart rate < 60 bpm) was observed in 12 patients (11.4%), and tachycardia (heart rate > 100 bpm) in one patient (0.95%). We did not observe any other significant arrhythmias. Median heart rate (IQR) was 76 (69–85) bpm. PR interval, QRS duration, and QTc are provided in Table 1.

First-degree atrioventricular block was observed in 2 patients (1.9%) with PR intervals of 205 and 235 ms. No higher degrees of atrioventricular block were detected.

QRS duration ≥ 120 ms was observed in 8 (7.7%) patients (maximum QRS width 152 ms). In this group, all patients were diagnosed with right bundle branch block; none of the patients had left bundle branch block or nonspecific intraventricular conduction delay. We did not observe QT interval (QTc) prolongation.

Echocardiography. The echocardiographic assessment of the study cohort revealed median values of measurements within the commonly accepted normal ranges for cardiac structure and function. Segmental wall motion was assessed in 99 patients, as 6 patients had image quality that precluded the determination of segmental wall motion. With each patient having 16 segments assessed, a total of 1584 segments were analyzed. Among the entire group, three segments were akinetic (0.19%), and 19 were hypokinetic (1.20%).

### 3.1. Comparison of Groups with Normal and Elevated Troponin I Levels

In fifty-seven (58%) patients, hs-TnI was not detectable (Trop− subgroup). Detectable levels of hs-TnI were observed in 42 (42%) patients (Trop+ subgroup). None of the analyzed groups had troponin levels exceeding the general population’s 99th percentile upper reference limit (15.6 pg/mL). 

The basic characteristics of subgroups according to the troponin levels are presented in Table 2. There were no differences in baseline characteristics between subgroups except for higher diastolic blood pressure in the troponin-positive subgroup (Trop+).

2D and Doppler echocardiography parameters are presented in Table 3. Patients in the Trop+ subgroup had larger left ventricular dimensions (4.7 vs. 4.9 cm, *p *= 0.02). They also had increased values for interventricular septum and posterior wall thickness (0.9 vs. 1.0 cm, *p *= 0.002 and 0.8 vs. 0.9 cm, *p *= 0.002, respectively). We also observed increased left ventricular end-diastolic and end-systolic volumes (101 vs. 109 mL, *p *= 0.004, and 38 vs. 45 mL, *p *= 0.01, respectively). However, LVEF did not differ between subgroups. We also noted significant differences in mitral inflow parameters and tissue Doppler indices. In the Trop+ subgroup, we observed higher values of the A wave velocity of mitral inflow (0.7 vs. 0.8 cm/s, *p *= 0.002) and lower E/A ratios (1.0 vs. 0.9, *p *= 0.004). We also found lower E wave velocities of tissue Doppler (12 vs. 10 cm/s, *p *= 0.002). 

### 3.2. Global Longitudinal Strain, Peak Systolic Dispersion, and Myocardial Work Indices

Speckle tracking-derived parameters are presented in Table 4. Patients Trop+ subgroup exhibited significantly worse global longitudinal strain (−18% vs. −19%, *p *= 0.008) and elevated PSD values (54 vs. 43 ms, *p* < 0.001)

The myocardial work indices for the entire study group were slightly below the recently proposed normal values [26]. In the Trop+ subgroup, we noted significantly higher values of GWW (168 vs. 97 mmHg%, *p* < 0.005) and lower GWE (93 vs. 95%, *p* < 0.005); however, GWI and GCW did not show significant differences. Regarding MW indices, no gender differences were observed for the whole group or when comparing the Trop− vs. Trop+ subgroups.

Intraobserver and Interobserver Variability. The ICC values for intraobserver and interobserver variability are presented in Table 5. The yielded values depicted a high reliability of the estimates about both intraobserver and interobserver across all the evaluated indices.

The correlation analysis revealed significant but weak-to-moderate relationships between myocardial work indices and the examined variables (Table 6). GWI correlated with LVEF (r = 0.35) mitral inflow E-wave velocity (r = 0.25, *p *= 0.01). Similarly, GCW correlated with LVEF (r = 0.32, *p *= 0.002). GWE was linked with LVEF (r = 0.24, *p *= 0.01) and the E/A ratio (r = 0.22, *p *= 0.03. Additionally, GWW was correlated with the diastolic dimension of the left ventricle (r = 0.24, *p *= 0.02).

Notably, we observed a moderate positive correlation between GWW and PSD (r = 0.63, *p* < 0.005) and negative correlations between GWI and PSD (−0.20, *p *= 0.05) and GWE and PSD (r = −0.63, *p* < 0.005).

We found significant correlations only for hs-TnI and MW indices among all evaluated laboratory parameters. The analysis revealed a significant negative correlation between hs-TnI and GWE (r = −0.41, *p* < 0.005) and a positive correlation with GWW (r = 0.41, *p* < 0.005). 

We constructed regression models for GWW and GWE (Table 7). The dataset incorporated clinically significant variables from our initial correlation analysis, including LVIDd, LVEF, E/A ratio, PSD, and hs-TnI levels. Each model was adjusted for confounders, such as age, BMI, time since recovering from COVID-19, and D-dimer and creatinine concentrations.

For GWW, only PSD was shown to be a strong independent predictor (β = 0.67, *p* < 0.001; R = 0.72, R² = 0.51, *p* < 0.005). For GWE, PSD emerged as the primary significant predictor (β = −0.67, *p* < 0.001, R = 0.73, R² = 0.53, *p* < 0.001), with LVEF also being a significant predictor (β = 0.16, *p *= 0.05).

## 4. Discussion

### 4.1. Summary of Findings

Our study aimed to assess myocardial work indices and hs-TnI levels in recently recovered COVID-19 patients. One of the main findings was that recovered patients who experienced mild to moderate COVID-19 exhibited decreased values of GWE, GCW, and GWI alongside increased GWW. Furthermore, in a subgroup of patients with increased hs-TnI levels, we observed significantly higher GWW and lower GWE but not GWI and GCW. Notably, this group also exhibited elevated hs-TnI concentrations below the decision level (99th percentile) but above the detection threshold. Ultimately, we demonstrated that PSD substantially accounts for the variability of myocardial work indices.

Although myocardial work indices have been the subject of some publications in COVID-19 patients [7,16,17,18,27,28], the available literature remains relatively scarce. Furthermore, there is no comprehensive exploration of these indices in the context of this disease. Our study thus addresses a significant gap in the existing research by examining the short to medium-term cardiac effects of COVID-19. This period is critical as it may present a window for intervention to prevent the progression to Long COVID-19, characterized by persistent symptoms and complications. For instance, literature focusing on hospitalized patients typically highlights acute cardiac manifestations and immediate outcomes [28,29], while studies involving patients beyond three months post-infection explore chronic conditions, ongoing myocardial inflammation, or persistent cardiovascular symptoms [18,27,30].

### 4.2. High-Sensitivity Troponin I in Our Study Group

In our study, we analyzed a group of patients where hs-TnI levels were either undetectable (below the detection level) or detectable but below the 99th percentile for the general population. The significance of detecting even low levels of troponin is supported by Than et al. [31]. The authors investigated the long-term risk associated with hs-TnI and high-sensitive Troponin T (hs-TnT) levels in patients presenting with possible acute coronary syndrome. They found that patients with detectable yet low levels of hs-TnI or hscTPT, below the upper reference limit, still faced an increased risk of major adverse cardiovascular events (MACE) and all-cause mortality over a five-year follow-up. Specifically, hazard ratios for MACE at the upper reference limit were 2.3 for hs-TnI and 1.8 for hs-TnT. Moreover, hazard ratios for all-cause mortality were 1.7 for hs-TnI and 2.3 for hs-TnT. These findings suggest that even troponin levels deemed negative for acute events could indicate a heightened long-term risk, emphasizing the importance of monitoring and addressing subclinical myocardial injury. These findings underline the relevance of including hs-TnI measurements in our study, even when levels are below the traditional threshold for acute myocardial infarction.

Similarly, Hayama et al. [32] investigated myocardial damage in recovering COVID-19 patients using hs-TnT levels and echocardiography. In their cohort of 209 recovered patients, they found that 35.4% had hsTnT levels below the detection sensitivity (<3 pg/mL), while 64.6% had hsTnT levels ≥3 pg/mL. Their study demonstrated that even slight increases in hsTnT above the detection sensitivity were associated with decreased left ventricular global longitudinal strain, indicating subclinical myocardial injury. The results of Hayama et al. parallel our results, where detectable but low levels of hs-TnI were associated with significant alterations in myocardial work indices, such as increased GWW and decreased GWE, suggesting that myocardial damage at these levels is clinically relevant.

### 4.3. Importance of Myocardial Work Indices

Myocardial work indices, including GWI, GCW, GWE, and GWW, provide valuable insights into cardiac function and efficiency post-COVID-19. Normal values of these indices are still under investigation [26,33,34,35,36]. Truong et al. [26] recently reported mean values for GWI 2010 mmHg% (95% CI, 1907–2113 mmHg%) and GCW 2278 mmHg% (95% CI, 2186–2369 mmHg%), with relatively narrow confidence intervals. In contrast, in our study group, we observed a median GWI of 1834 mmHg%, below the normative mean, and a median GCW of 2130 mmHg%, slightly under the established average. Furthermore, GWW in our study, with a median of 119 mmHg%, substantially exceeds the norm reported by Truong et al. of 80 mmHg% (95% CI, 73–87 mmHg%). These increased GWW values indicate a higher inefficiency in myocardial energy utilization, which could indicate persistent myocardial dysfunction or incomplete recovery in the post-COVID-19 context. Similarly, the GWE from our findings showed a median of 94%, slightly below the norm of 96.0% (95% CI, 96–96%).

The observed increased GWW and decreased GWE among patients with increased hs-TnI levels warrants careful consideration, particularly in COVID-19 infection. Elevated GWW indicates inefficiency in myocardial energy use, where a significant portion of the myocardial energy expenditure does not contribute to effective blood ejection, reflecting suboptimal cardiac mechanics [37]. Hs-TnI is a well-known biomarker of myocardial injury, and its elevation in COVID-19 patients has been linked to direct viral injury and heightened systemic inflammation [38,39]. The myocardial damage reflected by hs-TnI elevation could impair myocardial fiber function, reducing the efficiency of mechanical work performed by the heart and thus increasing GWW.

D’Ávila et al. [27] investigated longitudinal strain and myocardial work in symptomatic patients recovered from COVID-19, finding associations with disease severity. They assessed longitudinal strain and myocardial work indices in symptomatic patients who had recovered from COVID-19. However, unlike our study, the authors evaluated patients with long COVID-19 who had been recruited from a rehabilitation program. The authors observed an increase in GWW in the subgroup that experienced a critical course of the disease compared to the moderate/severe subgroup (GWW 146 (104–212) vs. 121 (74–163) mmHg%, *p *= 0.01). They also found that GWE was lower in this subgroup (93 (91–95) vs. 94 (93–96), *p *= 0.03). Illness severity was the only independent predictor of both GWW and GWE. Despite the observed differences in the studied populations, our study and D‘Ávila et al. reported changes in GWW, which may indicate a similar, persistent mechanism of myocardial abnormalities long after recovering from COVID-19.

Olsen et al. [28] explored the role of myocardial work indices in patients hospitalized with COVID-19, focusing on its relationship with biomarkers, disease severity, and all-cause mortality. They found that myocardial work indices, especially GWI, were significantly altered in patients with severe COVID-19 compared to those with milder forms of the disease. They found also that reduced GWI was significantly associated with increased disease severity and higher mortality rates. Their study linked these changes directly to the severity of COVID-19 and observed a correlation with increased levels of cardiac biomarkers similar to hs-TnI, which were also noted in our study. This correlation emphasizes the potential of myocardial work assessments as indicators of cardiac stress and injury in COVID-19 patients. Our study expands on these findings by highlighting that the severity of COVID-19 affects myocardial work indices and leads to an increase in GWW, indicating inefficiencies in myocardial energy use. The elevated GWW in our cohort, particularly among those with higher troponin levels, suggests that the myocardial involvement observed in severe COVID-19 cases may lead to substantial disruptions in cardiac mechanics.

### 4.4. PSD as an Indicator of Left Ventricular Contraction Inhomogeneity

Our study comprehensively analyses the relationships between myocardial work indices and various echocardiographic parameters in patients recently recovering from COVID-19. Notably, we observed moderate positive correlations between GWW and PSD (r = 0.63, *p* < 0.005), as well as a significant negative correlation between GWE and PSD (r = −0.63, *p* < 0.005). To our knowledge, this is the first study to describe the relationships between PSD and myocardial work indices (GWW and GWE) in patients who have recently recovered from COVID-19. In addition to the correlations between PSD and myocardial work indices, our study identified several significant relationships between myocardial work indices and conventional echocardiographic parameters. GWI, GCW, and GWE showed significant correlations with LVEF (r = 0.35, 0.31, and r = 0.24 respectively). These findings are consistent with the results of Zhu et al. [40], who also demonstrated significant correlations between myocardial work indices and conventional echocardiographic parameters, including LVEF in patients with coronary artery disease and heart failure. 

Furthermore, GWI was correlated with mitral inflow E-wave velocity (r = 0.25), while GWE was linked with the E/A ratio (r = 0.22). Moreover, the Trop+ subgroup had significantly worse diastolic function indices (A-wave 0.7 (0.6–0.8) vs. 0.8 (0.7–0.9) cm/s, *p *= 0.004, E/A Ratio: 1.0 (0.8–1.2) vs. 0.8 (0.7–1.0), *p *= 0.01 and E′: 12 (10–15) vs. 10 (8–13) (cm/s), *p *= 0.002). These associations suggest that changes in myocardial work due to SARS-CoV-2 infection are closely linked to diastolic function, reflecting the heart’s ability to relax and fill properly. Our results are in line with findings from other studies. Italia et al. [15] assessed 123 patients who recovered from COVID-19 and found a higher grade of diastolic dysfunction in patients with documented myocardial injury during hospitalization. Similarly, Tudoran et al. [41] found diastolic dysfunction in 16.8% of 125 patients assessed 6 to 10 weeks after acute COVID-19. Our results align with these studies, highlighting the prevalence and significance of diastolic dysfunction in the post-COVID-19 population.

However, after conducting regression analysis, incorporating significantly correlated parameters of left ventricular morphology, systolic and diastolic function, and potential confounders, PSD remained the main significant independent variable.

For GWW, PSD emerged as the only significant independent predictor (β = 0.67, *p* < 0.001). This finding underscores the pivotal role of myocardial synchrony in contributing to wasted myocardial work in patients recovering from COVID-19.

In the case of GWE, PSD was identified as the primary significant predictor (β = −0.67, *p* < 0.001), indicating a strong relationship between myocardial synchrony and cardiac work efficiency. LVEF showed a smaller but significant relationship with GWE (β = 0.16, *p *= 0.05), suggesting that while myocardial synchrony is a major determinant of work efficiency, overall left ventricular function also contributes.

Our results highlight the critical role of myocardial synchrony in modulating cardiac efficiency and wasted work in this population, providing new insights into the cardiac sequelae of COVID-19. Myocardial injury is extensively documented in the context of COVID-19 [2,29,38,42]. Based on the results of our study, it can be concluded that one of the elements of myocardial dysfunction following COVID-19 is heterogeneous contraction, which is reflected by abnormal values of PSD. PSD is an echocardiographic parameter that quantifies the temporal variability in myocardial strain across different cardiac segments during systole derived from speckle-tracking echocardiography. PSD is calculated as the standard deviation of time to peak systolic strain across the segments of the left ventricle. It reflects the degree of mechanical dyssynchrony within the myocardium, which can indicate underlying pathophysiological conditions across a spectrum of diseases, including heart failure [43], diabetes mellitus [44], hypertrophic cardiomyopathy [45], and left bundle branch block [46]. 

Several mechanisms could explain the increased GWW and decreased GWE observed in this study and the correlations with PSD. Firstly, COVID-19 has been associated with cardiac involvement, including myocarditis, which can disrupt myocardial fiber structure and function, leading to inefficient contraction and relaxation dynamics [47]. The inflammatory state induced by COVID-19 could exacerbate this by increasing myocardial stress and altering load conditions, thereby enhancing the proportion of wasted work [48]. However, in our study, we did not observe significantly elevated levels of inflammatory markers such as CRP, which had a median value of 1.1 (IQR: 0.6–2.8) mg/L, suggesting that the myocardial inefficiencies observed may not be solely driven by ongoing systemic inflammation, but rather the effect of direct myocardial injury and its sequelae. Furthermore, the laboratory data for our cohort showed median values of 6.9 × 10^9^/L for white blood cells, 4.6 × 10^12^/L for red blood cells, hemoglobin at 8.9 mmol/L, and hematocrit at 42.6%. Platelet counts were 253 × 10^9^/L, creatinine was 0.8 mg/dL, and eGFR was 88.3 mL/min/1.73 m^2^. These findings are indicative of a relatively stable inflammatory and hematologic profile post-recovery. The normal levels of inflammatory markers further support that the increased GWW and decreased GWE might be attributed more to direct myocardial damage rather than systemic inflammation.

COVID-19 has been closely associated with increased risks of thrombotic events, which are manifestations of a hypercoagulable state observed in this group of patients [49,50]. The D-dimer levels in our cohort had a median of 334.2 ng/mL (IQR: 247.2–529.6). While the median D-dimer level is within the normal range (less than 500 ng/mL), the interquartile range indicates that a significant portion of patients had elevated D-dimer levels. This suggests that, although the median value is normal, there is evidence of heightened thrombotic activity in a subset of patients. This hypercoagulable state could contribute to microthrombi formation, impairing myocardial perfusion and exacerbating myocardial dysfunction [51].

In addition to these mechanisms, recent research has highlighted the role of microRNAs as critical regulators of cardiovascular pathology in COVID-19 patients [52]. MicroRNAs have emerged as valuable biomarkers and predictors of both cardiac and vascular damage during SARS-CoV-2 infection. Specific microRNAs are involved in the modulation of inflammatory responses, myocardial injury, and thrombotic processes, all of which are relevant to the observed changes in myocardial work indices in our cohort. The identification of microRNAs as contributors to cardiovascular complications underscores the potential for these molecules to serve as early biomarkers and therapeutic targets, providing insights into the underlying molecular mechanisms driving increased GWW and decreased GWE in post-COVID-19 patients. Integrating microRNA analysis into future studies could enhance our understanding of the complex interactions between viral infection and cardiovascular dysfunction, ultimately guiding more effective interventions and management strategies for affected patients.

### 4.5. Limitations

This study has a few limitations. One obvious limitation is the lack of a control group. However, one of the aims of our study was to assess the significance of myocardial injury; thus, we compared two subgroups based on troponin levels. Another limitation of our study is the relatively small number of patients. This may explain the lack of statistically significant differences observed between the Trop+ and Trop− subgroups. This underpowering constrains our conclusions for GWI and GCW, pointing out the need for further study with a larger cohort to validate these findings. Despite this limitation, the sample size was adequate to detect significant differences in other parameters such as GLS, PSD, GWW, and GWE, thereby supporting the overall validity of these findings. Additionally, we did not manage the patients in the analyzed group during the active phase of COVID-19. Thus, we do not have echocardiographic data from the acute phase of the disease. Moreover, our study only provides a snapshot of myocardial work indices post-recovery. It does not include longitudinal follow-up data, which limits our ability to conclude the long-term cardiac effects of COVID-19 and the potential for these indices to predict long-term outcomes. The absence of pre-COVID-19 echocardiographic data for the patients makes it difficult to determine whether the observed myocardial work abnormalities were pre-existing conditions or directly related to the COVID-19 infection. The study cohort consisted of patients available for outpatient follow-up within three months post-recovery, which may introduce selection bias as those with more severe or prolonged illness may have been less likely to participate. Additionally, being a single-center study, our findings may have limited generalizability to other populations and healthcare settings. Lastly, unmeasured confounding factors, such as comorbidities and variations in treatment received during the acute phase of COVID-19, could influence myocardial work indices and were not accounted for in our analysis.

## 5. Conclusions

Our study comprehensively analyses the relationships between myocardial work indices and various echocardiographic parameters in patients recently recovering from COVID-19. The findings underscore the importance of myocardial synchrony in modulating cardiac efficiency and wasted work in the population. By integrating pressure-strain loop analysis into clinical practice, we can better understand the cardiac pathophysiology associated with COVID-19 and improve strategies for managing long-term cardiac complications post-infection. Elevated GWW and decreased GWE could be markers of post-infection myocardial injury, allowing for identifying patients more prone to developing long COVID, thus requiring special attention and possibly earlier treatment. The observed correlation between hs-TnI levels and GWW/GWE suggests a closer relationship between myocardial injury and myocardial work efficiency.

## Figures and Tables

**Table 1:** Basic characteristics of the study group.

Number of Patients	102
Gender: Male/Female (%)	45 (44.1)/57 (55.9)
Age (years)	52 (43.0–63.0)
BMI (kg/m^2^)	27.2 (23.4–29.9)
Systolic blood pressure (mmHg)	131.5 (122.0–143.0)
Diastolic blood pressure (mmHg)	82.0 (75.0–89.0)
Resting heart rate (beats/min)	76.0 (69.0–85.0)
Days since COVID-19 diagnosis	56.0 (42.0–71.0)
Number of pts hospitalized due to COVID-19 (%)	26 (26.0)
Comorbidities (%)	
Hypertension	36 (35)
Diabetes mellitus	10 (10)
Asthma/Chronic obstructive pulmonary disease	9 (9)
Ischemic heart disease	3 (3)
Laboratory data (n = 99)	
White blood cells (10^9^/L)	6.9 (5.8–8.4)
Red blood cells (10^12^/L)	4.6 (4.3–4.9)
Hemoglobin (mmol/L)	8.9 (8.2–9.5)
Hematocrit (%)	42.6 (39.8–45.6)
Platelet (10^9^/L)	253.0 (223.0–282.0)
D-Dimer (ng/mL)	334.2 (247.2–529.6)
Creatinine (mg/dL)	0.8 (0.8–0.9)
eGFR (mL/min/1.73 m^2^)	88.3 (79.6–97.4)
C-reactive protein (mg/L)	1.1 (0.6–2.8)
High-sensitive troponin I (pg/mL)	1.6 (1.6–2.7)
NT-proBNP (pg/mL)	60.4 (34.0–97.6)
Electrocardiographic Parameters (n = 104)	
PR interval (ms)	157 (140–173)
QRS duration (ms)	97 (90–105)
QTc interval (ms)	429 (412–443)
Echocardiographic data (n = 102)	
Interventricular septum thickness (cm)	0.9 (0.9–1.1)
Left ventricular internal diameter (cm)	4.8 (4.6–5.1)
Left ventricular posterior wall thickness (cm)	0.8 (0.7–0.9)
Left atrium dimension (cm)	3.7 (3.2–3.8)
Mitral E-wave velocity (m/s)	0.68 (0.6–0.78)
Mitral valve deceleration time (ms)	183 (150–208)
Mitral A-wave velocity (m/s)	0.73 (0.61–0.84)
Mitral valve E/A ratio	0.94 (0.75–1.14)
E′ (m/s)	0.12 (0.09–0.14)
E/E′	5.73 (4.72–7.32)
Left atrium volume index (mL/m^2^)	28.2 (23.1–31.4)
Left Ventricular Ejection fraction (%)	61 (58–64)
LVEDV (mL)	104 (86–123)
LVESV (mL)	41 (33–49)

Continuous data are presented as median (IQR), and categorical data as numbers (%). Of the 102 patients, three refused blood sampling. Abbreviations: eGFR—estimated glomerular filtration rate, NT-proBNP—N-terminal pro-b-type natriuretic peptide, E′—Tissue Doppler Mitral Annular E′ Velocity; E/E′—Ratio of mitral valve E-wave velocity to tissue Doppler Mitral Annular E′ Velocity, LVEDV—Left Ventricular End-Diastolic Volume, LVESV—Left Ventricular End-Systolic Volume.

**Table 2:** Subgroup characteristics based on high-sensitive troponin I levels.

	Normal Levels of Troponin I (n = 57)	Increased Level of Troponin I (n = 42)	*p* Value
Gender: Male/Female (%)	26 (45.6)/31 (54.4)	18 (42.9)/24 (57.1)	0.78
Age (years)	50.0 (38.0–64.0)	54.0 (43.0–61.0)	0.40
BMI (kg/m^2^)	27.3 (23.9–29.3)	26.7 (23.3–31.6)	0.56
Systolic blood pressure (mmHg)	130.0 (123.0–143.0)	133.5 (119.0–143.0)	0.73
Diastolic blood pressure (mmHg)	80.0 (74.0–87.0)	85.0 (78.0–90.0)	0.05
Resting heart rate (beats/min)	75.0 (68.0–85.0)	77.0 (70.0–83.0)	0.59
Days since COVID-19 diagnosis	56.0 (42.0–67.0)	57.5 (41.0–71.0)	0.75
Number of pts hospitalized due to COVID-19 (%)	13 (22.8)	11 (26.2)	0.70
Comorbidities (%)			
Hypertension	20 (35.1)	15 (35.7)	0.95
Diabetes mellitus	5 (8.8)	5 (11.1)	0.69
Asthma/Chronic obstructive pulmonary disease	6 (10.5)	3 (7.1)	0.56
Ischemic heart disease	2 (3.5)	1 (2.4)	0.75

Continuous data are presented as median (IQR) and categorical as numbers (%). Abbreviations: BMI—body mass index.

**Table 3:** Comparison of echocardiographic parameters between subgroups with normal and elevated levels of high sensitive Troponin I.

	Normal Levels of Troponin I (n = 57)	Increased Level of Troponin I (n = 42)	*p* Value
Interventricular Septum thickness (cm)	0.9 (0.8–1.0)	1.0 (0.9–1.1)	0.002
Left Ventricular Internal Diameter (cm)	4.7 (4.5–5.0)	4.9 (4.7–5.2)	0.009
Left Ventricular Posterior Wall thickness (cm)	0.8 (0.7–0.9)	0.9 (0.8–1.0)	0.002
Left Atrium Dimension (cm)	3.5 (3.1–3.8)	3.7 (3.4–3.9)	0.09
Mitral E-wave Velocity (m/s)	0.7 (0.6–0.8)	0.7 (0.6–0.8)	0.64
Mitral Valve Deceleration Time (ms)	178.0 (154.0–197.0)	201.0 (150.0–221.0)	0.14
Mitral A-wave Velocity (m/s)	0.7 (0.6–0.8)	0.8 (0.7–0.9)	0.004
Mitral Valve E/A Ratio	1.0 (0.8–1.2)	0.8 (0.7–1.0)	0.01
E′ (cm/s)	12 (10–15)	10 (8–13)	0.002
E/E′	5.4 (4.6–6.6)	6.3 (5.0–7.7)	0.07
Left Atrial Volume Index (mL/m^2^)	26.0 (22.4–30.4)	28.8 (24.4–35.9)	0.11
Left Ventricular Ejection fraction (%)	62.0 (59.0–65.0)	60.0 (55.0–64.0)	0.20
LVEDV(mL)	101 (83–116)	109 (96–130)	0.04
LVESV (mL)	38 (32–47)	45 (38–52)	0.01

Data presented as median (IQR). Abbreviations: E′—Tissue Doppler Mitral Annular E′ Velocity; E/E′—Ratio of mitral valve E-wave velocity to tissue Doppler Mitral Annular E′ Velocity, LVEDV—Left Ventricular End-Diastolic Volume, LVESV—Left Ventricular End-Systolic Volume.

**Table 4:** Speckle Tracking Echocardiography-Derived Parameters: Global Longitudinal Strain, Peak Strain Dispersion, and Myocardial Work Indices for the Entire Group and Subgroups Stratified by Troponin Levels.

	Entire Group (n = 97)	Normal Levels of Troponin I (n = 57)	Increased Level of Troponin I (n = 37)	*p* Value
GLS (%)	−19.0 (−20.0–−17.0)	−19.0 (−21.0–−18.0)	−18.0 (−19.0–−16.0)	0.008
PSD (ms)	45 (39–56)	43 (36–49)	54 (43–64)	<0.001
GWI (mmHg%)	1834 (1168–2054)	1857 (1867–2045)	1867 (1641–2060)	0.53
GCW (mmHg%)	2130 (2010–2398)	2120 (1976–2035)	2182 (2030–2460)	0.67
GWW (mmHg%)	119 (78–175)	97 (69–132)	168 (121–196)	<0.005
GWE (%)	94 (92–96)	95 (94–96)	93 (90–94)	<0.005

Data presented as median (IQR). The discrepancy between the total group size (n = 97) and the sum of the Trop(−) and Trop(+) subgroups arises from overlapping exclusions: outlier troponin I concentrations (3 patients) and poor echocardiographic image quality (6 patients), which precluded the analysis of GLS, PSD and MW indices. Abbreviations: GLS, Global Longitudinal Strain; PSD, Peak Strain Dispersion; GWI, Global Work Index; GCW, Global Constructive Work; GWW, Global Wasted Work; GWE, Global Work Efficiency.

**Table 5:** The Intraclass Correlation Coefficient for Intraobserver and Interobserver Variability.

Parameter	Intraobserver ICC (95% CI)	Interobserver ICC (95% CI)
PSD	0.93 (0.82–0.97)	0.90 (0.76–0.96)
GLS	0.97 (0.93–0.99)	0.89 (0.75–0.96)
GCW	0.98 (0.96–0.99)	0.94 (0.84–0.98)
GWE	0.83 (0.57–0.93)	0.81 (0.51–0.93)
GWW	0.85 (0.62–0.94)	0.84 (0.60–0.94)
GWI	0.96 (0.88–0.98)	0.88(0.62–0.95)

Abbreviations: ICC—intraclass correlation coefficient, CI—confidence interval, PSD: Peak Systolic Dispersion, GLS: Global Longitudinal Strain, GCW: Global Constructive Work, GWE: Global Work Efficiency, GWW: Global Wasted Work, GWI: Global Work Index.

**Table 6:** Correlation Coefficients between Myocardial Work Indices and Selected Cardiovascular Parameters.

	GWI	GCW	GWW	GWE
Troponin I	ns	ns	0.40; *p* < 001	−0.41; *p* < 001
PSD	−0.20; *p *= 0.05	ns	0.63; *p* < 0.001	−0.64; *p* < 0.001
LVEDd	ns	ns	0.24; *p *= 0.02	ns
LVEF	0.35; *p* < 001	0.32; *p *= 0.001	ns	0.25; *p *= 0.02
E wave	0.25; *p *= 0.01		ns	ns
E/A	ns	ns	ns	0.22; *p *= 0.03

Abbreviations: GWI: Global Work Index, GCW: Global Constructive Work, GWW: Global Wasted Work, GWE: Global Work Efficiency, LVEF: Left Ventricular Ejection Fraction, E/A: Ratio of Early (E) to Late (A) Mitral Inflov Velocities, LVEDd: Left Ventricular End-Diastolic Diameter, PSD: Peak Strain Dispersion, ns: Not Significant.

**Table 7:** Regression Analysis Results for GWW and GWI with Selected Parameters.

	Independent Variable	β (Beta)	SE (Standard Error)	t	*p*	R^2^
GWW	Age	−0.02	0.08	−0.25	0.80	0.51
	BMI	0.03	0.08	0.32	0.75	
	Time since infection	0.07	0.08	0.89	0.38	
	hs-TnI	0.09	0.09	1.02	0.31	
	D-Dimer	0.09	0.08	1.09	0.28	
	Creatinine	−0.14	0.08	−1.70	0.09	
	LVIDd	0.08	0.08	1.05	0.30	
	LVEF	−0.06	0.08	−0.78	0.44	
	E/A ratio	0.14	0.09	1.60	0.11	
	PSD	0.67	0.09	7.82	<0.001	
GWE	Age	0.01	0.08	0.08	0.94	0.53
	BMI	0.02	0.08	0.24	0.81	
	Time since infection	−0.08	0.08	−0.98	0.33	
	hs-TnI	−0.04	0.09	−0.52	0.60	
	D-Dimer	−0.12	0.08	−1.51	0.13	
	Creatinine	0.14	0.08	1.66	0.10	
	LVIDd	0.00	0.08	0.04	0.97	
	LVEF	0.16	0.08	1.99	0.05	
	E/A ratio	−0.08	0.09	−0.89	0.38	
	PSD	−0.67	0.08	−7.96	<0.001	

Abbreviations: β: standardized regression coefficient, SE: standard error of the standardized regression coefficient. t: t-statistic for the significance test of the regression coefficient. *p*: *p*-value, R²: coefficient of determination for the regression model (for the entire model), GWW—global wasted work, GWI—global work index, BMI—body mass index, hs-TnI—high-sensitive troponin I, LVIDd—left ventricular internal diameter at diastole, LVEF—left ventricular ejection fraction, PSD—peak systolic dispersion.

## Data Availability

The data supporting the reported results in this study are available from the corresponding author upon reasonable request. Due to privacy and ethical restrictions, the data are not publicly accessible. For further details, please contact the corresponding author.
